# The promise and peril of universal health care

**DOI:** 10.1126/science.aat9644

**Published:** 2018-08-24

**Authors:** David E. Bloom, Alexander Khoury, Ramnath Subbaraman

**Affiliations:** 1 Department of Global Health and Population, Harvard T. H. Chan School of Public Health, Boston, MA 02115, USA; 2 Center for Global Public Health and the Department of Public Health and Community Medicine, Tufts University School of Medicine, Boston, MA 02111, USA

## Abstract

Universal health care (UHC) is garnering growing support throughout the world, a reflection of social and economic progress and of the recognition that population health is both an indicator and an instrument of national development. Substantial human and financial resources will be required to achieve UHC in any of the various ways it has been conceived and defined. Progress toward achieving UHC will be aided by new technologies, a willingness to shift medical tasks from highly trained to appropriately well-trained personnel, a judicious balance between the quantity and quality of health care services, and resource allocation decisions that acknowledge the important role of public health interventions and nonmedical influences on population health.

**BACKGROUND:**

The September 1978 Alma- Ata Declaration is a landmark event in the history of global health. The declaration raised awareness of “health for all” as a universal human right,whose fulfillment reduces human misery and suffering, advances equality, and safeguards human dignity. It also recognized economic and social development and international security as not only causes, but also consequences, of better health. In addition, it highlighted the power of primary health care and international cooperation to advance the protection and promotion of health in resource-constrained settings.

Building on the achievement of Alma-Ata and gaining further traction from the Millenium Development Goals and the Sustainable Development Goals set by the United Nations, universal health care (UHC) has emerged in recent years as a central imperative of the World Health Organization (WHO), the United Nations and most of its member states, and much of civil society. UHC characterizes national health systems in which all individuals can access quality health services without individual or familial financial hardship. More broadly, UHC covers social systems that provide medical and nonmedical services and infrastructure that are vital to promoting public health.

**ADVANCES:**

Although there are numerous articulations of the UHC agenda, the WHO and World Bank offer a relatively simple UHC service-coverage index that is useful for intercountry comparison. This index focuses on four categories of health indicators: reproductive, maternal, and child health; infectious disease control; noncommunicable diseases; and service capacity and access. Comparison of UHC index values for 129 countries reveals that country index scores are positively correlated with income per capita, though there is considerable variation in scores among countries with similar incomes. These variations presumably reflect differentials in income inequality, commitment to public health infrastructure, and the quality and reach of human resources for health. The WHO and World Bank also offer multiple measures of health spending–related financial hardship in assessing UHC, which do not increase monotonically with increasing income, health spending per capita, or coverage of health services. Rather, catastrophic health expenditures tend to be lower in countries that channel health spending through public social security or insurance programs, rather than private insurance schemes.

**OUTLOOK:**

The financial cost of massively expanding access to health care globally is a formidable barrier to achieving UHC. For example, the Disease Control Priorities Network estimates that low- and lower-middle-income countries would, on average, need to raise their respective annual per capita health expenditures by U.S.$53 and U.S.$61 per person to achieve coveragewith the essential UHC package of 218 core interventions, a sizable burden in relation to average expenditure increases in recent years. Wealthy industrial countries are much further along the path to achieving UHC, though they also face challenges involving rising costs of new health care technologies and the growing share of their populations at the older (and more health care–intensive) ages.

Technically and economically efficient approaches to the achievement of UHC may include the use of electronic medical records, telemedicine systems, digital monitors for drug adherence, and clinical decision–support applications; expansion of the quantity and quality of human resources for health at the physician, nurse, and community health worker levels; improvements in inventory systems and supply chains for the delivery of vaccines, drugs, diagnostics, and medical devices; screening for risk factors and early signs of disease; and focusing on the often neglected domains of surgical care, reproductive health, and mental health. Also key will be efforts to ensure universal access to proven public health interventions that address social and environmental determinants of health, such as health education campaigns; access to safe water; regulation of excessive sugar and salt in the food supply; control of tobacco and the unsafe consumption of alcohol; road traffic safety; walkable city designs; expanding enrollment in high-quality primary and secondary schools; and more equitable distributions of income and wealth.

Achieving UHC is an ambitious aspiration and a powerful indicator of human progress. Fortunately, it may be expected to deliver myriad health, economic, and social welfare benefits along the way, helping to mobilize the substantial political and financial resources needed for its continued future expansion.

**Figure:**
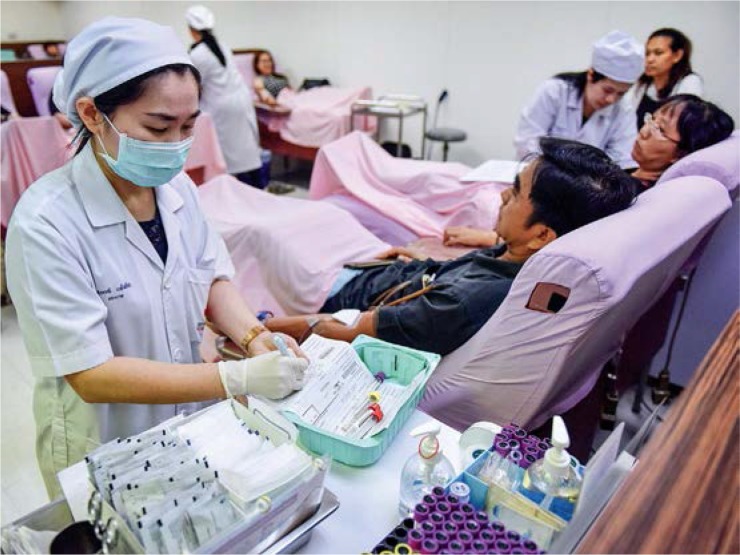
**Bangkok, Thailand.** A nurse keeps records of a blood donor at the Thai National Blood Centre on 14 June 2017.

Universal health care (UHC) characterizes national health systems wherein all individuals can access quality health services without individual or familial financial hard ship. More broadly, UHC covers social systems that provide medical and nonmedical services and infrastructure that are vital to promoting public health.

ThenotionofUHCdates toOtto vonBismarck, who established the world’s first national social health insurance system in Germany in 1883 (*1*). More recently, the September 1978 Alma-Ata Declaration raised global awareness of “health for all” as a universal human right and of the power of primary health care to advance its achievement (*2*). During the 20th century, many industrialized countries extended UHC to their citizens. Although progress in expanding UHC slowed in the 1980s—mainly because of economic slowdowns, fiscal stress, and structural adjustment programs—achieving UHC in all countries is currently among the central imperatives of the World Health Organization (WHO), the United Nations (UN) and most of its member states, and much of civil society.

As the Alma-AtaDeclaration’s 40th anniversary approaches, we examine the rationale, progress, consequences, and prospects for achieving UHC globally. We first explore the rationale for UHC, the scope of what UHC encompasses, and its operational definitions at the international level. We then report statistics on current measures of UHC attainment, highlighting patterns by country income level. We go on to review evidence on three key premises of UHC: that it promotes longer, healthier lives; that it does so efficiently; and that it confers social, economic, and political benefits above and beyond the utilitarian value of living healthier lives. Finally, we discuss prospects for further expansion of UHC.

We argue that UHC has considerable potential to improve the trajectory of human progress. To achieve UHC, however, governments and the public health community will have to mobilize substantial human, financial, and technological resources and avoid pitfalls in implementation.

## Rationale and scope

Four sets of arguments are commonly advanced in support of UHC. The first set appeals to ethics and morality and the notion that safeguarding everyone’s physical and mental health is just, fair, and consistent with principles of right conduct and distributive justice. The second argument, rooted in international law, relates to the acceptance of health as a fundamental human right (*3*). The third set of arguments is pragmatic, relating to the observation that healthy populations tend to be more socially cohesive and politically stable. The final set of arguments is economic in nature: UHC corrects health-related market failures, such as those related to the social benefits of disease prevention among individuals, and good health may promote economic well-being not just among healthy individuals but also at the macroeconomic level (*4*). These economic arguments are bolstered by evidence that committing resources to health care is associated with a high return on investment, rivaling, or even surpassing, other high-return investments like those in primary and secondary education (*5*–*8*).

Although there is a strong rationale for the possible benefits of UHC, there are also numerous challenges to its realization. A central challenge preceding any realization of UHC is defining its scope and boundaries. Although precise definitions of UHC vary widely among sources, the WHO’s definition is a typical formulation of the concept as a system in which “all individuals and communities receive the health services they need without suffering financial hardship. It includes the full spectrum of essential, quality health services, from health promotion to prevention, treatment, rehabilitation, and palliative care” (*9*).

This definition highlights many of the ambiguities involved in conceptualizing UHC. What levels of reduced mortality risk, increases to quality of living, or other thresholds must be crossed before a health service is considered needed or essential? Should financial hardship be defined by the amount of money spent relative to income, the amount of income that households retain after health spending, or some other criteria? Should these criteria shift or remain constant across settings? Given this definition’s emphasis on health services, does UHC also imply a commitment to addressing social and environmental health determinants beyond the traditional purview of health service delivery?

As discussed below, the answer to this last question may have considerable implications for UHC’s effectiveness in improving health outcomes. It is widely accepted that most health outcomes are associated with social and environmental factors, including wealth, income inequality, discrimination, education, occupation, diet, substance use, violence and conflict, air pollution, and water and sanitation access (*10*, *11*). Addressing these factors is central to emerging public health agendas such as One Health (which views human, animal, and environmental health holistically) and Planetary Health (which focuses on the economic and social systems that shape human and environmental health). Deficiencies in the availability and quality of medical services are important but, nonetheless, contribute less to premature mortality than these nonmedical determinants (*11*). But even though UHC definitions that address nonmedical health determinants have greater potential to improve health outcomes, operationalizing a UHC agenda that addresses these determinants would require wide-ranging interventions in sectors outside of health care, which may be more politically, socially, and technically challenging.

Given the ambiguities in defining UHC, there are several possible approaches to put the concept into practice. These approaches vary according to intended use, such asmaking comparisons across countries, tracking progress over time, or delineating a roadmap for achieving UHC. The WHO and World Bank offer a relatively simple UHC service-coverage index (hereafter, “the WHO– World Bank index”), which is useful for intercountry comparisons. They define this index in terms of 16 indicators, grouped into four categories: reproductive, maternal, newborn, and child health; infectious-disease control; noncommunicable diseases; and service capacity and access (*9*).

This relatively small number of indicators allows 129 countries to be included in the UHC service-coverage index. The indicators are meant to serve as a proxy for the overall coverage of the health care system, which should ideally provide many more health services than those represented.

However, although measurement of a handful of tracer conditions and services has often been used as a proxy for overall health system quality, many public health experts have concerns that only indicators that get measured actually get implemented in practice. In addition, the health services included in the index are fairly basic—in terms of the medical conditions covered, skill levels required by health care personnel, and technological capacity required—limiting this metric’s value for comparing high income countries with well-funded health systems. Some of the indicators, such as access to insecticide-treated bed nets for malaria prevention, have minimal relevance in most high-income countries. In addition, these indicators do not comprehensively capture many of the high burden diseases that could be successfully addressed with health services in high income countries, such as treatment for most types of cancer.

The WHO and World Bank also describe multiple approaches for measuring health spending–related financial hardship in assessing UHC. They suggest two thresholds for annual health spending— equal to 10 and 25% of total household expenditures—as alternative metrics for routinely measuring catastrophic health spending, which refers to out-of-pocket expenses exceeding a household’s ability to pay without imposing considerable financial hardship. Two additional metrics aim to more directly assess impoverishment resulting from health expenditures, by measuring the percentage of households whose average daily non health consumption expenditures would have placed its members above the U.S.$1.90 and U.S. $3.20 per capita poverty lines but for the household’s spending on healthcare (*12*). Given the very low thresholds for impoverishing health expenditures, these metrics are primarily relevant in low and middle-income countries (LMICs).

More comprehensive UHC priority descriptions exist. For example, the Disease Control Priorities (DCP) Network has compiled 218 distinct cost-effective interventions, which they argue should form a standard of essential services for LMICs because they address a substantial burden of disease. Unlike the indicators in the WHO– World Bank index, more than one-third of the DCP Network’s essential interventions—including tobacco taxes, air pollution reduction, and road safety improvements—focus on broader social or environmental determinants and would require non–health care sector involvement. (*13*). A subset of 108 interventions, termed the highest-priority package, avert death or disability while also scoring highly on a financial risk protection index. Comprehensive data are not available on population coverage for many interventions included in the DCP Network’s UHC package, limiting its use in making comparisons among countries. Measurement and inclusion of many of these evidence-based services should be considered in future iterations of the global UHC agenda. coverage increase with rising income levels (*14*), it is also likely that the higher country incomes are, at least in part, the result of better health care coverage and health (*4*). Disparities in UHC service coverage by income level are even more apparent when looking at groups of countries together: The average service-coverage score for low-income countries is roughly half that of high income countries (Table 1). Sub-Saharan Africa and South Asia feature the lowest index scores, whereas the Latin American and Caribbean and the East Asia and Pacific regions have index scores comparable to those in North America and in Europe and Central Asia.

**Table 1:** **Population, income, health expenditure, and UHC index score by income group and geographic region**. Figures are weighted according to population size. Source: World Bank (2018) (15), with UHC service-coverage index scores and catastrophic health expenditure data from World Bank (2017) (9). All data are for 2016, except for the health expenditure data, which are for 2015, and the catastrophic health spending data, which are for 2010.

	Number of countries	Percentage of world population (%)*	Income per capita (current U.S.$)	Health expenditure per capita (current U.S.$)	Health expenditure as a percentage of GDP (%)	Mean UHC index score (range)	Percentage of households experiencing catastrophic health spending (%)†
**World**	130	100	10,192	1,002	9.8	63 (29 to 80)	11.7
**Income group**							
Low income	21	8	616	35	5.7	39 (29 to 53)	8.1
Lower-middle income	39	42	2.078	83	4.0	53 (33 to 73)	12.4
Upper-middle income	35	36	7.994	470	5.9	74 (52 to 78)	13.8
High income	35	15	40.826	5.050	12.4	79 (64 to 80)	7.2
**Region**							
Sub-Saharan Africa	35	14	1.467	85	5.8	42 (29 to 67)	10.3
South Asia	7	26	1.638	58	3.5	53 (34 to 62)	13.5
Middle East and North Africa	11	4	7.200	416	5.8	64 (39 to 80)	13.4
East Asia and Pacific	14	32	9.783	626	6.4	72 (47 to 80)	12.9
Europe and Central Asia	44	12	22.238	2.089	9.4	72 (54 to 80)	7.0
Latin America and Caribbean	17	8	8.342	637	7.6	75 (57 to 79)	14.8
North America	2	5	56.102	9.031	16.1	80 (80 to 80)	4.6

* Percentage of world population refers to the entire income group or region, not just the countries included in the sample.

† Catastrophic health spending refers to the proportion of individuals in the population who live in households that spend >10% of their consumption expenditure on out-of-pocket health care costs (9).

Also notable are the instances of similar income countries having highly disparate index scores. For example, Nigeria and Vietnam both have per capita GDPs around U.S.$2200, but Vietnam’s UHC index score is 34 points higher than Nigeria’s. This reflects the fact that Vietnam outperforms Nigeria on several indicators, including reported rates of three-dose diphtheriatetanus- pertussis infant vaccination coverage (94 versus 42%), births attended by skilled professionals (94 versus 35%), and households with access to basic sanitation (78 versus 32%). Dissimilar income distributions in the two countries offer a plausible partial explanation for the coverage discrepancies. An estimated 78% of Nigeria’s population lives on less than U.S.$3.20 per day in 2017 dollars, compared with only 32% of Vietnam’s population (*15*, *16*). Poverty imposes constraints on accessing health services, particularly in LMICs (*17*). Furthermore, less-comprehensive health service coverage reinforces poverty by failing to protect individuals from illnesses that have high treatment costs or that limit their ability to work or learn (*18*).

**Fig. 1:**
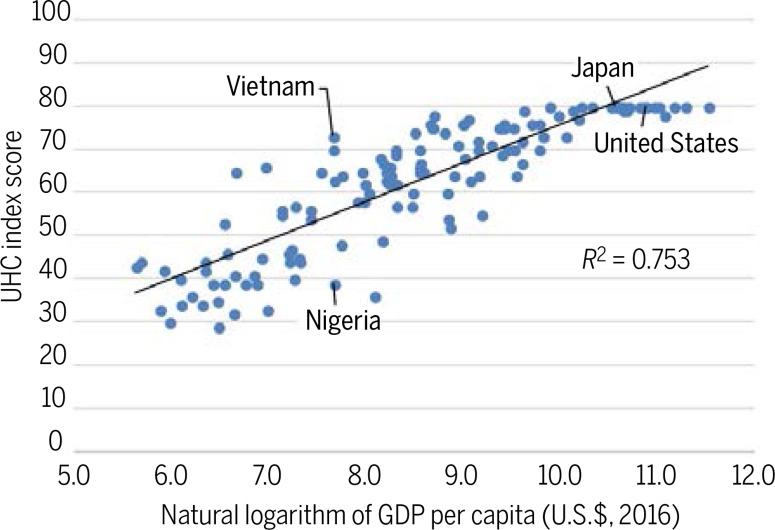
**WHO–World Bank UHC index score versus the natural logarithm of GDP per capita.** The plot captures absolute changes in UHC index scores (maximum of 80) relative to percentage changes in GDP per capita. Source: UHC index scores from World Bank (2017) (*9*) and GDP per capita from World Bank (2018) (*15*). R^2^, coefficient of determination.

Unlike the association between UHC service coverage and GDP per capita (Fig. 1), protection from catastrophic health expenditures is not clearly correlated with GDP per capita. In aggregate, middle-income countries have higher rates of catastrophic health expenditures than low- and high-income countries (Table 1). However, the variation in catastrophic expenditure rates within these income groups is greater than the variation among them. Furthermore, protection from catastrophic health expenditures does not systematically improve with increasing UHC service-coverage index score or with increasing percentage of GDP spent on health care (*19*). Thus, protection from health care–related financial ruin does not directly follow from GDP growth, improved essential health service coverage, or increased total health care spending. Rather, catastrophic health expenditures may be associated with the pathways through which health care spending occurs. Countries in which much of health spending is prepaid through public social security or insurance programs tend to have lower catastrophic health expenditure rates than countries that mostly rely on private insurance schemes (*19*).

Even among countries attaining the maximum UHC index score of 80, there is substantial heterogeneity in health outcomes, health spending, and the proportion of the population protected from catastrophic health spending. Comparing the UHC records of two high-income countries with perfect index scores, the United States and Japan, illustrates these disparities.

The United States is the only high-income country that does not explicitly provide UHC for its citizens, although its relative expenditures on health care—15% of GDP in 2008 and 17% of GDP in 2017—are much higher than those of any other Organisation for Economic Cooperation and Development country (*20*). Unlike the United States, Japan expanded health insurance coverage to its entire population in 1961. This change coincided with a massive improvement in the health of Japan’s population, which, by 1983, had the highest life expectancy of any country (and also now, at 84 years) (*15*). Japan’s health system has been lauded for its role in promoting a world-leading level of population health and for maintaining relatively low health care costs historically. However, these health expenditures have risen from 8% of GDP in 2008 to 11% in 2017 ( ), and the Japanese health care system must adapt to a continually increasing elder share of the population while constrained by an economy that has performed relatively poorly since the 1990s.

## Consequences of expanding UHC

The premise that UHC could lead to longer, healthier lives has a strong underlying rationale. For most indicators in the WHO–World Bank index, achieving high coverage could benefit individuals through reduced disability, increased longevity, improved nutritional status, increased economic productivity, or decreased health-related financial hardship (Table 2).

**Table 2:** UHC essential services as defined by the WHO and World Bank and the rationale for their impact on health and social outcomes.

Essential health service	Selected evidence for beneficial health, social, or economic outcomes
Family planning	Decreased maternal mortality (*64*), improved economic growth associated with reduced fertility (the “demographic dividend”) (*31*)
Antenatal and delivery care	Reduced infant and maternal mortality (*65*)
Child immunization	Reduced mortality for children less than 5 years old, improved educational attainment and economic productivity (*23*)
Pneumonia care	Reduced pneumonia-related morbidity and mortality (*66*)
Tuberculosis treatment	Improved tuberculosis treatment success, mortality, and prevalence (*22*, *45*, *67*)
HIV antiretroviral therapy	Increased life expectancy (*68*), reduced HIV transmission (*21*)
Insecticide-treated bed nets for malaria prevention	Reduced malaria episodes and child mortality (*69*)
Access to basic sanitation	Reduced mortality and stunting of children less than 5 years old (*70*)
Prevention and treatment of elevated blood pressure	Reduced cardiovascular and all-cause mortality in individuals more than 60 years old (*71*)
Prevention and treatment of elevated blood sugar	Reduced microvascular complications of diabetes, including kidney failure, loss of vision, and nerve damage (*72*)
Cervical cancer screening	Reduced cervical cancer incidence and mortality (*73*)
Tobacco (non)smoking	Reduction in lung cancer, obstructive pulmonary disease, cardiovascular, and all-cause mortality (*74*)
Basic hospital access	Lower maternal mortality (*28*), lower mortality from life-threatening emergencies (*75*)
Health care worker density	Reduced all-cause child and adult mortality and reduced health disparities among populations (*27*, *29*)
Access to essential medicines	Reduction in the proportion of the population experiencing catastrophic health care costs (*76*, *77*)
Compliance with international health regulations (health security)	Early detection of disease outbreaks (*78*), with benefits and limitations highlighted by the 2013–2014 Ebola outbreak (*79*)

Increased coverage of services can also have a population-level health impact, especially for leading infectious causes of death, such as tuberculosis (TB), HIV/AIDS, and malaria. For these diseases, early treatment of affected individuals can terminate the chain of transmission, thereby reducing disease incidence. For example, over a 7-year time period, HIV-uninfected individuals living in areas with high antiretroviral therapy coverage in Kwa Zulu Natal, South Africa, were 38% less likely to acquire HIV than those in areas with low coverage (*21*). Similarly, in China during the 1990s, TB prevalence declined only in provinces where the directly observed therapy short-course (DOTS) strategy—which involves provision of free or subsidized TB testing and treatment—was rolled out with high coverage (*22*).

Similarly, expanding vaccine coverage through the UHC agenda—especially for leading causes of child mortality such as Streptococcus pneumoniae, Haemophilus influenzae, and rotavirus—would have a population-level health impact in a highly cost-effective manner. The full societal benefits of disease prevention through vaccination include increased schooling and labor productivity, slowing of the pace at which antimicrobial resistance develops, and reductions in health and economic risk, all magnified by the value of improved health outcomes among non vaccinated community members owing to herd effects (*23*).

Regarding the potential impacts of UHC on both health and financial hardship, some compelling evidence is found in the Oregon Health Insurance Experiment (*24*). In 2008, the U.S. state of Oregon randomly selected about 30,000 individuals to be eligible to apply for Medicaid from among the roughly 90,000 who had expressed interest in applying to the newly expanded program providing low-cost health coverage for low-income adults. Through comparisons of individuals who were not selected to those who applied and were accepted, researchers found that receiving Medicaid virtually eliminated catastrophic medical spending, reduced medical debt, increased use of preventive medical care, reduced depressive symptoms, and improved subjective perception of overall health status. Measures of physical health—including control of high blood pressure, high cholesterol, and diabetes—did not significantly improve among individuals who received Medicaid; however, the 2-year follow-up time for individuals may have been too short to detect meaningful improvements in these outcome indicators.

Other literature on the impact of increased coverage and density of primary care and hospital based services on health outcomes is generally of weaker quality. Nevertheless, examples from Costa Rica and Cuba suggest a strong association between the universal expansion of public sector primary care services and rapid reductions in child and adult mortality and increases in life expectancy (*25*, *26*). In addition, a systematic review highlights the consistency, across a variety of LMIC contexts, of the positive association between large-scale primary care initiatives and lower child mortality (*27*). In other LMIC settings, increased hospital access is associated with reduced maternal mortality (*28*). In high-income countries, a higher density of primary care providers is associated with lower all-cause mortality (*29*).

Beyond improving health, expanding UHC could potentially promote economic well-being, reduce economic inequalities, and bolster social and political stability (*5*, *30*). Improving population health could accelerate economic growth by improving labor productivity, school attendance, educational attainment, cognitive function, capital accumulation, and fertility control (*31*, *32*). Rigorous microeconomic evidence supports the impact of health improvements on individual or House hold economic circumstances. Interventions with demonstrated effects on education and earnings include iodine supplementation (*33*), iron supplementation (*34*), deworming (*35*, *36*), and malaria eradication campaigns (*37*). These benefits may also have an appreciable macroeconomic impact (*38*): On average, a 10-year life expectancy gain is associated with up to a 1% increase in annual income per capita growth (*5*).

The impact of better health on economic growth may be particularly powerful in LMICs, where children, adolescents, and prime-age adults are the chief beneficiaries of health gains, leading to improvements in productivity across the life course (*37*). Ensuring access to basic health care, especially for the prevention and treatment of infectious diseases, may be essential for escaping poverty traps in settings where extreme poverty has historically been persistent (*39*, *40*). But benefits of health on economic growth are also manifest in high-income countries, where gains in longevity tend to accrue disproportionately to older adults. The social and economic value resulting from these gains in longevity for older adults may not be well represented in national GDP because the value created is often related to the enabling effect of health on nonmarket activities such as child-rearing, caretaking of other individuals, and community volunteer work (*41*).

Expanding UHC also reduces health disparities because poor members of society are less likely to receive adequate health care than wealthier individuals where UHC systems are lacking. Increased access to primary care is associated with reduced wealth- and race-based mortality disparities in both LMICs (*27*) and high-income countries (*29*). As noted, decreasing health inequality may also reduce income, wealth, and education disparities. As with investments in education, expansion of health care coverage is one of the rare policies that simultaneously promotes equitable distribution of income while also increasing economic growth (*6*, *42*). Reducing disparities through improved public health and social welfare systems may help to minimize the risk of political and social instability, though empirical evidence of this association is not especially robust (*43*). Through these various pathways, UHC serves important functions that support a healthy, prosperous, and cohesive society.

Although the potential benefits of UHC are numerous, possible pitfalls in implementation could undermine its impact and prevent UHC from fulfilling its promise. Rapid scale-up of UHC without sufficient concern for the quality of implementation could have unintended adverse consequences, as delivery of health services will not be effective in improving health outcomes if the delivered care is not of reasonable quality. Deficiencies in quality of care such as medical errors, spread of infection in health care settings, and poor retention of patients across sequential steps of care (also known as the cascade of care) could undermine the benefits of expanded service coverage. Even though existing UHC frameworks allude to this problem, quality-related indicators can be hard to measure, and achieving high quality of care will be especially challenging with large-scale expansion of coverage (*44*).

**Fig. 2:**
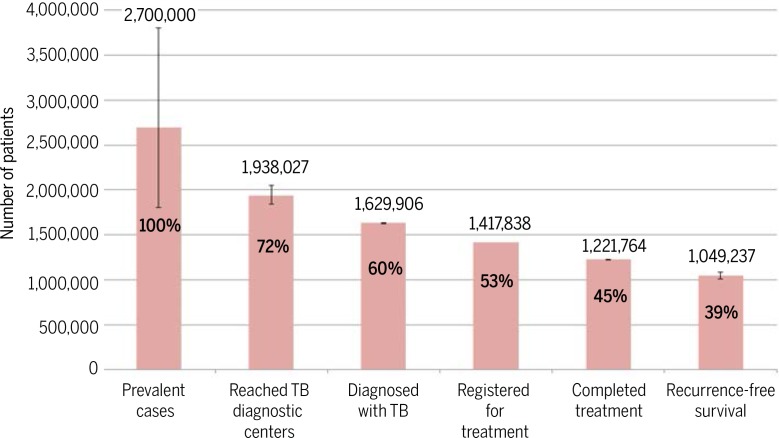
**Cascade of care for patients with any form of TB in India in 2013.** Patient losses at each stage of care represent shortcomings in quality of care that undermine the effectiveness of TB services, despite a high level of population coverage. Source: Subbaraman et al. (*47*).

The recent history of TB care delivery illustrates limitations of focusing on coverage of health services without ensuring that the services offered are of sufficient quality to be effective. In 1991, the World Health Assembly adopted the DOTS strategy, which included comprehensive coverage of free or subsidized TB testing and treatment as a key objective. Over the next two decades, high-burden countries such as India and China achieved high DOTS coverage nationally, leading to reductions in disease prevalence or TB-related mortality (*22*, *45*). However, despite high global DOTS coverage, TB incidence is declining slowly (<1.5% per year); the disease remains the leading infectious cause of death, resulting in nearly 1.7 million deaths annually, one-third of which occur in India. Poor quality of care may in part explain these disappointing public health outcomes (*46*). For example, in India, considerable numbers of patients are lost across sequential steps of the care cascade; as a result, only about 39% of prevalent TB patients were estimated to have achieved an optimal outcome in the government program in 2013 (Fig. 2) (*47*). Similarly, in Rwanda, improved rates of maternal institutional delivery have not translated into reductions in newborn mortality, likely owing to gaps in care quality (*48*).

As these examples suggest, poorly functioning health systems are a central challenge to realizing the benefits of UHC. Health systems in LMICs commonly suffer from a variety of weaknesses, including absenteeism and insufficient training among health care workers, mistreatment of patients by health care workers, corruption, poorly functioning inventory systems and supply chains, electricity cuts and outages, and lack of clean water. These shortcomings in health care delivery often reflect higher-level problems in governance and market failures. Achieving UHC will therefore require innovations in the structure and operation of health systems to ensure that rapid expansion in coverage is not undermined by shortcomings in delivery and quality of care.

With regard to the scope of UHC, it is entirely appropriate for countries to prioritize different health interventions in their UHC agendas to address local needs and constraints. It is also reasonable to expect the number of health services considered essential in each setting to undergo progressive expansion over time to reflect changing resource availability and to address new or emerging health concerns. For example, the WHO–World Bank index—perhaps the most prominent articulation of the UHC agenda— mostly focuses on health service coverage for conditions that have been long-standing global health priorities, such as maternal health, HIV, and TB. The index does not emphasize measurement of service coverage for other conditions that contribute substantially to the global burden of disability or death, such as depression and anxiety (the leading causes of disability globally) and conditions that require basic surgical care (inaccessible to about 5 billion people) (*49*, *50*).

In addition, the WHO–World Bank index includes a measure of access to essential medicines but does not cover access to essential diagnostic tests, which are crucial to address population level threats to health, such as antimicrobial resistance (*51*). Rising rates of antimicrobial resistance could be a major unintended consequence of UHC if increasing health care coverage does not go hand-in-hand with expanded access to diagnostic tests that facilitate judicious use of antibiotics. As suggested by these examples, if countries adhere to an overly narrow set of UHC priorities, they could miss out on opportunities to address conditions for which there is a dearth of health care providers and institutional capacity in LMICs.

The relatively limited inclusion of measures of nonmedical health determinants in most UHC frameworks represents another, more fundamental, limitation in scope. The WHO–World Bank index focuses on assessing delivery of medical services, with the exception of access to adequate sanitation and insecticide-treated bed nets. The UHC scope thus defined largely avoids the question of ensuring universal access to many public health interventions that could lead to healthier lives—including health education campaigns, in-home piped water supplies, regulation of excessive sugar and salt in the food supply, tobacco control, road traffic safety, construction of walkable cities, high quality primary and secondary education, and equitable distribution of wealth.

Two examples illustrate the limitations of a UHC approach that avoids addressing underlying nonmedical health determinants. In the United States, the dramatic rise in mortality among middle-aged white people in recent years occurred during a time of increasing health insurance coverage in the general population. These “deaths of despair”—largely attributable to mortality from substance use, suicide, and injuries—are thought to be driven by social determinants, such as lack of employment opportunities for blue-collar workers and increasing wealth inequality (*52*).

Another example is stunting owing to chronic child undernutrition, which is associated with poor health outcomes, cognitive development, and educational attainment. Most factors that contribute to stunting—poverty, lack of maternal education, poor maternal nutrition, lack of dietary diversity, and lack of sanitation—reflect failures to address nonmedical health determinants (*53*, *54*). In India, which accounts for 40% of the world’s stunted children, social inequalities such as gender and caste discrimination drive deficiencies in maternal education and sanitation access, thereby impeding progress in reducing stunting (*53*, *54*). As these examples suggest, UHC that narrowly focuses on health service delivery alone is necessary, but insufficient, to bring about wide-ranging health and social benefits. UHC will be implemented within the wider context of the Sustainable Development Goals (SDG) set by the UN, which includes targets related to some of these nonmedical determinants; however, embedding these SDG targets within a UHC-related public health framework could shape the approach and intensity with which these targets are achieved.

## Prospects

The financial cost of massively expanding access to health care globally is a formidable barrier to achieving UHC. The cost of attaining UHC partly hinges on a population’s existing health, which is influenced by factors such as age structure, levels of physical activity, pollution, water and sanita sanitation infrastructure, vaccination coverage, and diet. Using their broad operationalization of UHC described above, the DCP et work estimates that low- and lower-middle-income countries would, on average, need to raise their respective annual per capita health expenditures by U.S.$26 and U.S.$31 per person to achieve coverage with the highest priority package (108 core interventions); achieving coverage with the essential UHC package (218 core interventions) would require an annual spending increase of U.S.$53 and U.S.$61 per person on average (*13*).

However, the authors caution that achievement of even the essential UHC package would not be sufficient to reach the SDG target of reducing deaths of individuals less than 70 year sold by 40% by 2030. Achieving the highest-priority and essential UHC package would accomplish around half and two-thirds of this goal, respectively (*13*). Presumably, covering the essential health services in the WHO–World Bank index would require lower per capita health expenditure but would be expected to fall even shorter in reaching the SDG targets

As Table 3 shows, the health expenditure growth needed to achieve essential UHC in LMICs by 2030 is comparable to the rate of health spending increases that these countries experienced in recent years. However, these raw estimates of recent growth in health spending could paint an overly optimistic picture. A recent study from the Global Burden of Disease Health Financing Collaborator Network uses data from a similar period (1995–2015) and an ensemble of models that include covariates associated with GDP and health expenditure growth (such as fertility rates and mean years of education) to project health expenditure growth through 2030 (*55*). The Network projects that the difference between the number of individuals covered by UHC in the “worst-case” and “best-case” health financing scenarios would be about 871 million people (*55*).

**Table 3:** **Health expenditures needed to attain the highest-priority package (HPP) and essential UHC (EUHC) package by income.** Source: Watkins et al. (2017) (*13*), with public health expenditure data and average growth (2000–2015) calculated from WHO (2018) (*15*).

Health expenditure metric	Low-income countries	Lower-middle-income countries
Public health expenditures per capita (U.S.$)*	18	28
Total (and incremental†) health expenditure per capita needed for HPP (U.S.$)	42 (26†)	58 (31†)
Total (and incremental†) health expenditure per capita needed for EUHC (U.S.$)	76 (53†)	110 (61†)
Average annual growth rate in public health expenditures needed to achieve HPP by 2030 (%)‡	6.6	5.3
Average annual growth rate in public health expenditures needed to achieve EUHC by 2030 (%)‡	10.3	8.4
Average annual growth rate in real public health expenditures per capita 2000–2015 (%)*	9.8	9.2

* Values provided refer to government and donor health expenditures per capita in 2012 U.S.$.Table 1 provides total health expenditures for LMICs (including private expenditures).

† Incremental health expenditures per capita refers to the amount health spending per person would have to increase from current levels to support the complete package of interventions.

‡ The estimated growth in public health expenditures needed to achieve HPP and EUHC assumes that all additional coverage for these packages are met through government expenditure and that all additional government health care expenditure is spent on these intervention packages.

Given the sizable expenditure increases necessary to achieve UHC, rolling out UHC programsin stages will be necessary. The Lancet Commission on Investing in Health advocates a “progressive universalist” approach to funding these efforts, whereby selected health services are offered broadly and affordably to all citizens by the government, even if this necessitates offeringa smaller package of interventions. The authors argue that this approach is more efficient and equitable than a system that covers more interventions but necessitates higher out-of-pocket expenditures or restricts coverage to fewer individuals (*56*).

In light of expected health expenditure increases required to achieve UHC, physicians and public health practitioners may have to radically rethink strategies for health care delivery to simultaneously improve efficiency and health outcomes. For example, lack of trained health care personnel, especially in LMICs, is arguably the most serious hurdle to scaling up UHC (*57*). In many countries—such as India, Bangladesh, and Uganda—most health care personnel are informal providers who lack formal medical training (*58*). Informal providers are often assumed to deliver low-quality care; however, a recent randomized trial found that intensive training sessions with these providers can improve the quality of care that they deliver to a level that is, in some cases, on par with formal providers (*59*). Careful and constructive engagement with these informal providers may therefore be one strategy for bridging the substantial health care workforce gaps that threaten to undermine progress toward UHC in LMICs. Stemming outmigration of physicians from LMICs through bonding schemes (such as conditional scholarships) or enforcement of ethical recruitment policies in high-income countries may also help to reduce health care worker shortages (*60*).

Programs to recruit and train community health workers (CHWs) offer another, more widely accepted, strategy for expanding the health care workforce and increasing the coverage and effectiveness of primary health care. Growing evidence suggests that these programs can contribute to improved outcomes in child nutrition, maternal health, HIV, and TB (61). Moreover, CHW programs could potentially expand the reach of health care provision to the household level. This would be especially beneficial in the context of a rapidly increasing global burden of chronic disease. Primary and secondary prevention of chronic diseases requires early screening for risk factors and lifelong treatment of those risks (e.g., medications for hypertension), and many chronic diseases and risk factors cluster within households (*62*). CHWs may also have an important role in tracking newborns at the household level from the first to the last vaccination during infancy. By extending screening, monitoring, and treatment of medical conditions to the household level, CHW programs could have substantial effects on preventing disease, increasing rates of health screening, and improving treatment outcomes, thus improving UHC coverage, efficiency, and impact.

Integrating innovative technologies into health systems—including electronic medical records, clinical decision–support applications, telemedicine, digital medication-adherence technologies, and point-of-care diagnostic tests—could also facilitate UHC by improving the reach, timeliness, efficiency, and quality of clinical care and public health monitoring. These technologies could improve the quality and coverage of longitudinal clinical records, facilitate health care providers’ use of evidence-based clinical care algorithms, extend access to specialized medical knowledge to rural communities, reduce time delays for diagnosis and treatment, and enable real-time monitoring of medication adherence. Artificial intelligence and machine learning have the potential to perform some tasks—such as interpreting x-rays, electrocardiograms, and electroencephalograms— that currently require highly trained and specialized health care workers.

Technological innovations will not obviate the need to dramatically increase the health care workforce in LMICs, but they could still prove to be game changers as the global community tries to rapidly scale up health service delivery to achieve UHC. The ambitious scope of the UHC agendamay provoke physicians and public health experts to reimagine how to deliver health services. New frontline health care personnel (such as CHWs and nonhealth professionals receiving appropriate training) and innovative technologies could help to move care provision into nontraditional spaces, such as homes or workplaces, extending the existing health system’s effective reach.

## The bottom line

Four decades after the Alma-Ata Declaration articulated primary care for all as being a most important worldwide social goal, the global community is striving to achieve UHC with renewed interest and ambition. A central motivation of the UHC agenda is the belief that access to health care—with the goals of extending longevity, minimizing disability, and diminishing suffering—is a fundamental human right that advances equality and safeguards human dignity. Achieving UHC would represent one of the most ambitious ventures in the area of human rights, even if UHC were defined narrowly as universal delivery of essential health services. In addition, evidence suggests that well-implemented universal coverage of essential health services could improve welfare more broadly, by reducing economic inequalities, promoting economic well-being, and, perhaps, improving social and political stability.

A broader UHC conception that aims to also address the nonmedical determinants that most strongly shape human health would have even greater implications for society and would require broader social transformations. Addressing cross-cutting social and environmental determinants that contribute to ill health—such as wealth inequality; race, gender, and caste discrimination; air pollution; and lack of water and sanitation facilities—could lead the UHC agenda to intersect more closely with the human rights, One Health, and Planetary Health agendas in the coming decades.

Numerous potential pitfalls could impede UHC expansion or undermine its positive impact on health and well-being. Most challenging, perhaps, is the need to increase health financing rapidly enough to facilitate universal coverage of essential health services among LMIC populations that are simultaneously growing in size and aging. For example, for the world’s less-developed regions, an increase of roughly 1 billion people isprojected from 2018 through 2030, with the percentage aged 60 years or older projected to increase from 10.6 to 14.2%(*63*). In addition, a UHC agenda that fails to address social determinants of health could limit its impact on health outcomes. Finally, focusing too much on coverage alone, rather than on ensuring the quality of health services, ould undermine UHC effectiveness. Addressing these challenges may require radical transformations in the way that health services are delivered, potentially by expanding the use of frontline health personnel and incorporating innovative technologies into care delivery.

Ultimately, the path to UHC and the interventions prioritized in this process will be unique to each country pursuing universal coverage. Although achieving full UHC is a daunting task, incremental steps toward fulfilling this goal also offer myriad health, economic, and social welfare benefits. cognizing these benefits should help mobilize the resources needed for continued future expansion of UHC.
